# Driving forces of the pervasiveness of street vending: A data article

**DOI:** 10.3389/fpsyg.2022.959493

**Published:** 2022-09-12

**Authors:** Salem A. Al-Jundi, Sarah Basahel, Abdullah S. Alsabban, Mohammad Asif Salam, Saleh Bajaba

**Affiliations:** ^1^School of Business, Skyline University College, Sharjah, United Arab Emirates; ^2^Department of Management Information Systems, King Abdulaziz University, Jeddah, Saudi Arabia; ^3^Department of Business Administration, King Abdulaziz University, Jeddah, Saudi Arabia; ^4^Department of Business Administration, Faculty of Economics and Administration, King Abdulaziz University, Jeddah, Saudi Arabia

**Keywords:** poverty, unemployment, education, immigration, urban culture, consumption, resistance, microfinance

## Abstract

Street vendors are prominent on public streets and in traditional markets in most developing countries. They raise significant problems for public authorities, residents, pedestrians, and formal retailers. Their informal business is problematic, leading to conflicts and sometimes violence. Moreover, unlicensed street vendors employ children and women and are accused of counterfeiting and drug trading. However, they participate in reducing poverty and unemployment. The current data article aims to formulate a public perception on the problematic issue of street vending pervasiveness by describing a survey dataset on street vending and its main driving factors. Street vending has traditionally be examined by linking it with one or more determinants; thus, the dataset covers poverty, lack of education, immigration, unemployment, urban culture, low-income consumption, resistance, and lack of microfinance as latent constructs. Five measurable variables are introduced that reflect each construct. All variables are measured via seven-point Likert scales. Using a Google Form, 425 responses were collected that reflect the attitudes of the general public in Baghdad, Iraq. This dataset is useful for research on socio-economic problems; more specifically, it introduces reliable measurement models for street vending and the eight factors driving it.

## Introduction

**FIGURE 1:**
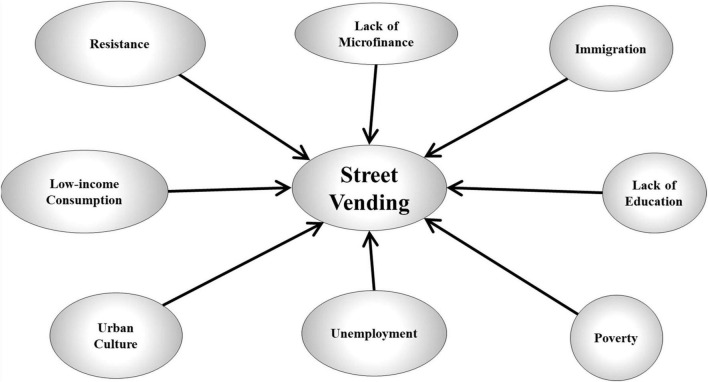
Conceptual model of street vending.

Street vendors (SVs) occupy public streets and traditional markets in most developing countries. Their informal business is problematic for public authorities, residents, pedestrians, and formal retailers, leading to conflicts, and sometimes violence. Additionally, unlicensed SVs employ children and women and are accused of counterfeiting and drug trading (Ilahiane and Sherry, 2008). However, they participate in reducing poverty and unemployment. Furthermore, by offering cheap goods and services to low-income customers, they can provide an important function in poor agricultural societies (Martínez et al., 2018). Research on street vending has suggested several factors driving this form of business (Figure 1); these include poverty; lack of education; immigration; unemployment; urban culture; low-income consumption; resistance; and lack of microfinance (Voiculescu, 2014; Tamilarai and Angayarkanni, 2016; Alvi and Mendoza, 2017; Wibisono and Catrayasa, 2018). This data article seeks to establish measurement models for these constructs prior to designing an electronic survey. These models were tested for reliability. The sample collected reflects the perception of the general public in Baghdad, the capital of Iraq.

Researchers have previously interviewed SVs to investigate vendors’ characteristics and driving forces influencing their livelihood, while other studies have used published national data on a macro level. The current study follows the suggestion raised by Chai et al. (2011). For this reason, this study seeks to formulate the perception of ordinary people on the problematic issue of the pervasiveness of street vending in Baghdad as an initial step toward revisiting public policies on how to fairly deal with the problem which affects the daily life of cities in developing countries such as Iraq.

Street vending is a historical phenomenon in most Iraqi cities, including Baghdad. Public authorities most likely accept the existence of the informal sector within traditional markets and on public streets. However, the municipalities sometimes evict peddlers and demolish their stalls.

Iraq has suffered successive wars since the 8-year Iraq–Iran war that began in 1980. The United States-led alliance occupied the country in 2003, and military and security forces were dismantled and demobilized. Thus, political stability has been increasingly damaged, and financial resources allocated to fighting terrorism, sectarian tension and extremists, and tribal fighting. Such circumstances have led to adverse events: (1) the unemployment rate has increased sharply, and the state has stopped recruiting fresh graduates and youth as it had done during Saddam Hussein’s dictatorial regime, (2) government entities have weakened and people do not respect the law, and (3) poverty has increased among a large proportion of the population. As a sequential result, the number of SVs has significantly increased due to poverty, unemployment, immigration from cities affected by terrorism, and dropouts from schools. Moreover, the country’s infrastructure is weak, and the government has been unable to carry out further development. By occupying confined and underdeveloped public spaces, SVs have become a significant problem for citizens, pedestrians, formal sellers, and public authorities. Therefore, it is important to deepen understanding of this phenomenon by formulating a public opinion about it.

It should be noted that SVs are somewhat entrepreneurs attempting to escape from poverty and unemployment. In general, the informal sector participates in eliminating unemployment and poverty and contributes significantly to Iraq’s gross domestic product. SVs (also known as peddlers or hawkers), increase the vibrancy of cities, although their role is mostly unappreciated. They usually work in an unsafe environment and suffer irrational purges from traditional markets and sidewalks.

Street vendors seek to maximize their profits regardless of the needs of municipalities, pedestrians, citizens, and their counterparts in the formal sector. Also, they extensively compete with each other and with formal sellers in overcrowded markets. Furthermore, they overrun the narrow spaces in front of shops and do not care about traffic flow and the underdeveloped infrastructure. And, they discard their waste in public spaces. Although they work many hours daily and have no paid vacation, they do not have the financial resources to transition into formal businesses. They are also characterized by: (1) they share similar features with home-based enterprises, (2) they can be young or elderly, single or married, women or men, and educated or not, and (3) they try to develop their skills by responding to customers’ changing needs. Licensed SVs who establish close ties with public authorities should be distinguished from unlicensed ones who struggle due to eviction campaigns and lack basic infrastructures such as electricity or a water supply. The current survey dataset attempts to explore the factors affecting unlicensed SVs.

Street vending and the main factors driving it are considered latent variables or constructs. To measure them and study their relationships and interconnections, it is necessary to establish measurement models for all constructs. The current paper suggests reflective measurement models by introducing five reflective indicators for each construct. All observed items were measured using seven-point Likert scales (1: strongly disagree to 7: strongly agree). To the best of our knowledge, previous literature has not provided measurement models or observed indicators to measure constructs because the concepts are ambiguous.

## Experimental design, materials, and methods

### Procedure

The measurable variables of street vending and the main factors driving it (see Tables 1–9), were translated into Arabic, the native language in Iraq. The items were then uploaded as a Google Form for an e-survey. Thereafter, we discussed the context of the questionnaire with five academic faculties in the Middle Technical University, Baghdad, and invited 25 friends and relatives to complete the survey. This process led to fine-tuning of the questions to match the culture and context of Baghdad.

**TABLE 1:** Measurement properties of street vending.

Codes	Items	Mean	Median	Standard deviation	Skewness	Outer loadings
SV1	SVs spread through the streets and markets of the city.	5.205	6	2.229	−0.941	invalid
SV2	City authorities do not provide any support to SVs.	5.461	6	1.992	−1.139	0.648
SV3	SVs seek to improve their income without paying attention to the traffic in the street and the movement of pedestrians on the pavements.	5.751	7	1.822	−1.569	0.8
SV4	SVs compete with each other in the overcrowded streets and markets.	5.769	6	1.71	−1.569	0.769
SV5	SVs are unlicensed by the municipality or other governmental offices.	5.734	7	1.764	−1.42	0.757

**TABLE 2:** Measurement properties of poverty.

Codes	Items	Mean	Median	Standard deviation	Skewness	Outer loadings
PO1	Most poor families are large.	5.645	6	1.476	−1.317	0.709
PO2	Agricultural production has declined over the last decade.	6.179	7	1.405	−2.049	0.74
PO3	SVs cannot expand their business.	5.024	5	1.823	−0.7	0.558
PO4	Teenagers, younger children, and girls earn money in the streets.	5.76	6	1.666	−1.604	0.733
PO5	SVs suffer dirt, noise, abuse, and health hazards.	5.998	7	1.547	−1.914	0.78

**TABLE 3:** Measurement properties of lack of education.

Codes	Items	Mean	Median	Standard deviation	Skewness	Outer loadings
LE1	SVs are almost illiterate or have little education.	4.647	5	1.823	−0.48	0.896
LE2	SVs rarely have adequate qualifications to apply for a job in the formal sector.	4.52	5	1.931	−0.315	0.562
LE3	SVs rarely have a university degree.	4.384	5	1.961	−0.282	0.588
LE4	School dropout rates have increased over the last decade.	6.045	7	1.396	−1.84	0.83
LE5	Schools and universities do not properly equip people to work in modern companies.	5.28	6	1.782	−0.933	0.687

**TABLE 4:** Measurement properties of immigration.

Codes	Items	Mean	Median	Standard deviation	Skewness	Outer loadings
IM1	Some SVs come from areas that have experienced war or conflicts.	5.296	6	1.634	−0.926	0.761
IM2	Some SVs come from rural areas.	4.859	5	1.765	−0.64	0.8
IM3	Some SVs come from poor areas.	5.464	6	1.582	−1.133	0.797
IM4	Women and their children who come from other areas work in the streets.	5.205	5	1.583	−0.879	0.794
IM5	Some SVs, who come from other areas, have succeeded in setting up successful businesses in the streets.	4.976	5	1.66	−0.699	0.573

**TABLE 5:** Measurement properties of unemployment.

Codes	Items	Mean	Median	Standard deviation	Skewness	Outer loadings
UN1	The unemployment rate among youth and fresh graduates is very high.	6.353	7	1.26	−2.748	0.814
UN2	There is no unemployment compensation.	5.913	7	1.579	−1.695	0.802
UN3	The duration of unemployment is very long.	5.981	7	1.457	−1.906	0.858
UN4	The rural sector does not offer jobs.	5.664	6	1.667	−1.427	0.717
UN5	Street vending is a current source of employment.	5.694	6	1.555	−1.451	0.619

**TABLE 6:** Measurement properties of urban culture.

Codes	Items	Mean	Median	Standard deviation	Skewness	Outer loadings
UC1	My city is vibrant despite the spread of SVs.	4.485	5	1.927	−0.474	0.713
UC2	Some SVs offer traditional and delicious food.	4.713	5	1.899	−0.595	0.726
UC3	Some SVs prefer working in the streets rather than in the formal sector.	4.699	5	1.923	−0.623	invalid
UC4	People enjoy walking and communicating in the traditional markets.	5.104	5	1.653	−0.862	0.77
UC5	People can find interesting books in a special traditional market.	5.885	6	1.351	−1.595	0.742

**TABLE 7:** Measurement properties of low-income consumption.

Codes	Items	Mean	Median	Standard deviation	Skewness	Outer loadings
LC1	SVs are so close to my home.	3.8	4	1.974	0.006	invalid
LC2	Goods, such as fruit and vegetables, are somewhat cheaper on the streets than in shops.	5.492	6	1.56	−1.209	0.756
LC3	SVs offer delicious and cheap cooked food.	4.504	5	1.742	−0.469	0.798
LC4	I can find souvenirs and accessories at low prices in the streets.	5.214	5	1.555	−0.888	0.856
LC5	SVs offer similar goods to shops.	4.871	5	1.687	−0.686	0.669

**TABLE 8:** Measurement properties of resistance.

Codes	Items	Mean	Median	Standard deviation	Skewness	Outer loadings
RE1	SVs resist being evicted from sidewalks and traditional markets.	5.668	6	1.473	−1.383	0.751
RE2	SVs have developed strategies to enable them to stay in the streets.	5.652	6	1.408	−1.315	0.821
RE3	SVs will return to their sites if the city’s officials demolish their stalls.	5.647	6	1.485	−1.357	0.786
RE4	SVs sometimes protest against their eviction from the streets.	5.605	6	1.422	−1.274	0.745
RE5	SVs occupy certain markets or sidewalks depending on their social networks.	5.701	6	1.397	−1.406	0.8

**TABLE 9:** Measurement properties of lack of microfinance.

Codes	Items	Mean	Median	Standard deviation	Skewness	Outer loadings
LM1	SVs cannot get formal credit facilities from commercial banks.	5.784	6	1.536	−1.405	0.774
LM2	SVs depend on their savings and selling family assets to start micro-businesses.	5.478	6	1.549	−1.145	0.741
LM3	If SVs manage to get a loan, they pay an exorbitant interest rate.	5.398	6	1.645	−1.044	0.753
LM4	SVs cannot afford the rent of retail outlets.	4.995	5	1.802	−0.652	0.684
LM5	There is no specialist public organization to financially support micro-businesses.	5.139	6	1.832	−0.766	0.637

Some important discoveries were made at this stage. Public officials tend to ignore SVs rather than evict them from the sidewalks of traditional markets because of the high pervasiveness of street vending and the abnormally high unemployment rate. Surprisingly, some SVs hold educational qualifications, including secondary school certificates, diplomas, and even undergraduate degrees, which can be attributed to the extremely high unemployment among youth and recent graduates. Additionally, most peddlers are Iraqis, with a minority from the neighboring country of Syria, which has struggled with severe conflicts and terrorism. We had assumed that lots of Syrian migrants might have fled to Baghdad. However, we found that Syrians have fled to Kurdistan in northern Iraq, which enjoys better stability and economic conditions than the rest of Iraq, as well as to Turkey and Europe. Most have not emigrated to Baghdad because the Iraqi capital has suffered from terrorism and conflicts. Tables 1–9 show the final results of the questionnaire.

To obtain complete answers to all 45 statements within the limited resources of this research, the questionnaire was distributed online via social media, such as Facebook and WhatsApp, as well as direct emails to our friends in the city. Previous literature has reviewed the SVs themselves to understand their characteristics and the environment in which they work (Reid et al., 2010). There is still an urgent need to review the attitudes of the general public to the issue of street vending. This procedure is a necessary step prior to revisiting public policies on how to deal with the problem, particularly with respect to considering the two sides of the issue: peddlers have negative effects on traffic flows, the environment, tax collection, and the competition with the formal sector; however, they have positive effects by reducing poverty and unemployment (Chai et al., 2011). The current dataset reflects the public perspective in Baghdad. To reach participants, the survey was published online, and we personally invited students, administrative staff, and academic faculties of the Middle Technical University to complete the form. Additionally, we encouraged students and other friends to promote the survey among their contacts, including their friends on social media. This process helped to reach a reasonable number of participants from different backgrounds and across the entire capital city.

We aimed to gather 600 responses that reflect the perception of different social classes in Baghdad. Because of limitations of time and resources, we successfully collected only 463 complete answers (response rate around 77%), taking into consideration that, the Google Form did not allow incomplete surveys to be submitted. After screening the dataset, 38 (8%) responses were found to be unreliable: these respondents answered the 45 statements with similar choices for each. The standard deviation of responses of each of these participants was close to zero, so these answers were omitted. Therefore, the final sample size is 425 complete and usable responses. Data collection took 3 months from the beginning of September to the end of November 2018. The dataset is fairly a good presentation of public attitudes toward the high pervasiveness of street vending in Baghdad. This dataset can be viewed and downloaded online through the Mendeley Data repository at Al-Jundi (2019). This study applies non-probabilistic sampling with a large population (Baghdad’s 2021 population is estimated at more than seven million), and the sample of 425 responses should be considered small in the context of the total population of the capital city of Iraq. The findings would be more accurate if we could increase the sample so that it is more representative of the population of Baghdad as a whole.

### Demographic profile of participants

**FIGURE 2:**
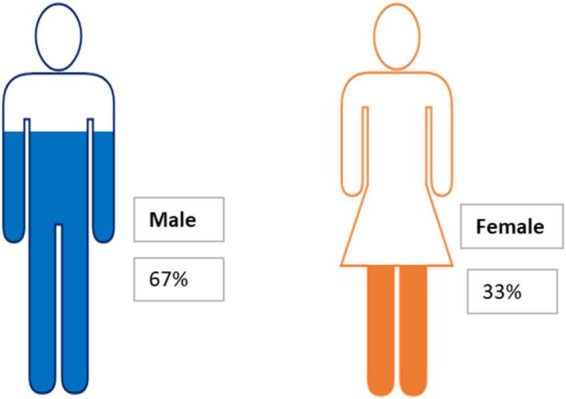
Gender distribution of the 425 participants.

**FIGURE 3:**
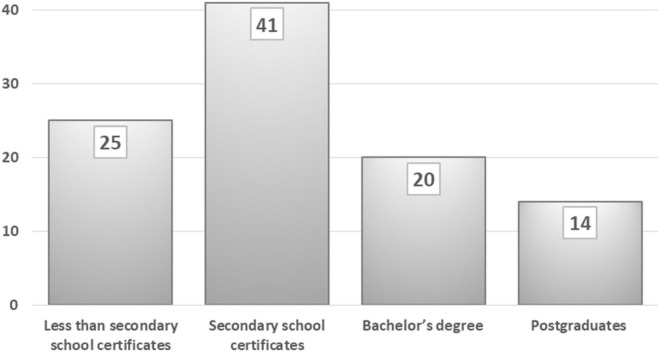
Highest educational level of the 425 participants (%).

Although we initially invited members of the university community, the online mechanism and our students helped to reach people from different backgrounds. As a result, the sample of 425 responses is quite diversified. Figure 2 shows that 67% of participants are male and 33% are female. Due to social and cultural constraints in Baghdad, men are freer and more active than women, since the latter cannot express their opinions as freely in the virtual world. Figure 3 presents the educational attainment of participants, 25% of whom do not have secondary certificates, with some of them dropping out of schooling at different ages. I could not reach illiterate people through the Internet even though they are an important category in society. Of the sample, 41% hold secondary school certificates; some are university students who show significant interest in public issues; 20% of participants hold bachelor’s degrees and work in the public and private sectors; and 14% represent academic faculties from different universities in Baghdad and show concern about research and academic surveys. The educational attainment across the sample may not coincide with that of society as a whole, but the variety of educational levels gives a fair reflection of society.

**FIGURE 4:**
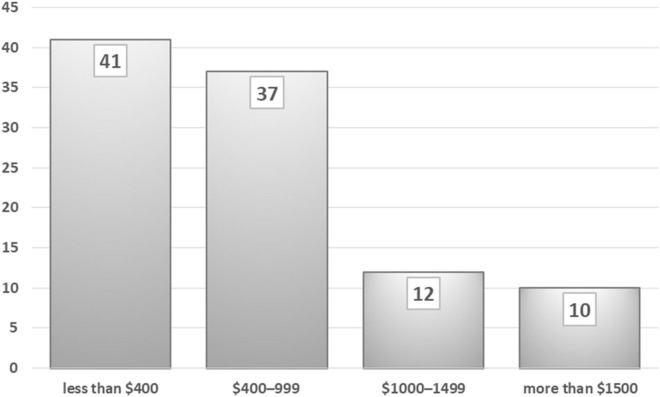
Monthly household income of the 425 participants (%).

**FIGURE 5:**
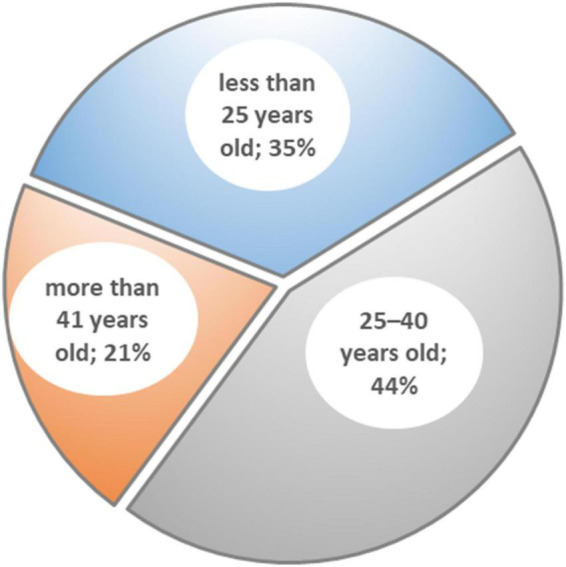
Age distribution of the 425 participants.

Figure 4 presents the division of household monthly income: 41% of participants earn less than US$400; 37% have an income in the range US$400–999; 12% earn US$1,000–1,499; and 10% bring home more than US$1,500. The distribution of the sample is reasonably representative of the allocation of income across the city. Figure 5 depicts the age distribution of the sample: 35% of participants are under 25 years old; 44% are 25–40 years old; and 21% are more than 41 years old. The age distribution is varied and shows that young adults are more interested than older people in public issues. The sample is quite representative of the society of Baghdad and it is not biased. The dataset can be used for further analysis to examine the relationships and interplay between street vending and the eight factors driving it. Measurable models can be applied to understand this issue in other cities.

## Data analysis and results

### Street vending

The existence of street vending can be tracked by indicators, such as its pervasiveness; the degree of competition among SVs; SVs’ relationship with public authorities; and the extent to which SVs are unlicensed and careless of traffic flows.

To deepen understanding of the behavior (pattern) of observed variables, we can compare the mean (average), median (center), and mode (most frequent). Which of the mean, median, and mode represents the central tendency? If the data are graphed symmetrically or takes the shape of the normal distribution, the three parameters will be close to each other. Let us check whether an indicator in the dataset has a normal distribution (bell curve) or is skewed (shifted) to the right or left. In our case, the observed variables are most probably negatively skewed. The curve is shifted to the right and has a flatter (longer) tail to the left side of the data distribution. Consequently, the mean will be lower than the median, and the latter will be lower than the mode. Thus, the mean is strongly affected by low scores and does not fairly represent the central tendency. The mode is somewhat extreme. Thus, the median reasonably reflects the center of the dataset of an indicator.

**FIGURE 6:**
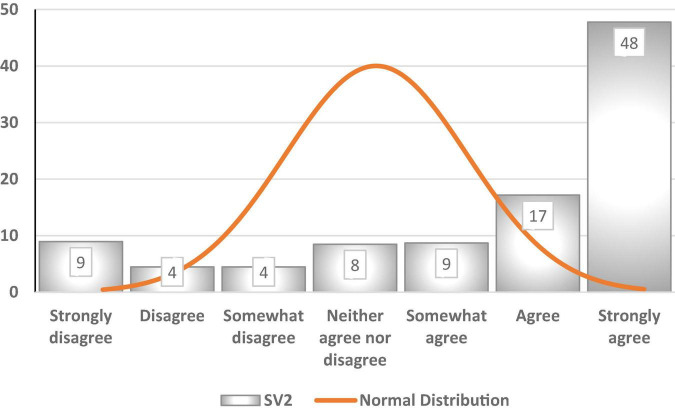
Histogram of SV2 (%).

**FIGURE 7:**
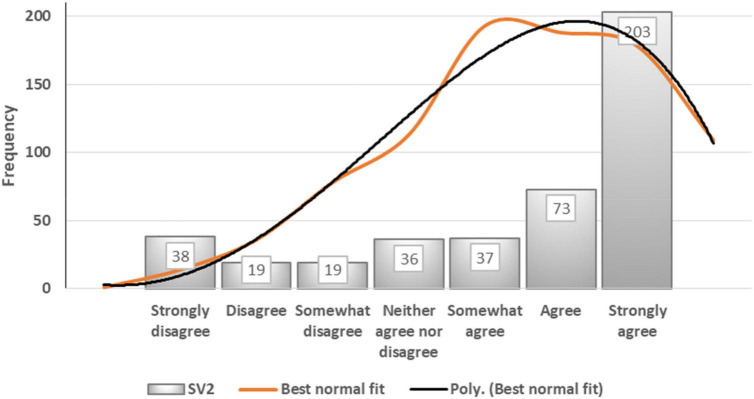
Best normal fit curve of SV2.

Figure 6 depicts a street vending indicator referred to as SV2 (“City authorities do not provide any support to SVs”). Among participants’ responses to this statement, 48% selected “strongly agree,” 17% chose “agree,” and 9% selected “somewhat agree.” The mean (5.5) is lower than the median (6), and the latter is lower than the mode (7). The dataset for this indicator is far from being a bell curve or normal distribution. Figure 7 shows that the data are negatively skewed – that is, the best normal fit curve shifts to the right and has a longer tail on the left side. The median (6) can be said to represent the central tendency, namely that 74% of the sample of 425 participants agreed with the statement.

This study used SmartPLS version 3 to calculate all parameters in the tables (Ringle et al., 2015). For socio-economic problems such as street vending, it is recommended to use partial least squares structural equation modeling (PLS-SEM) rather than a covariance-based SEM because the former does not require normal distribution assumptions whereas the latter requires normality of the dataset (Fornell and Bookstein, 1982).

Table 1 summarizes the properties of the five measurable variables of street vending. The mean is much higher than “5: somewhat agree” and is close to “6: agree.” The mode is at “7: strongly agree” about the existence of street vending in Baghdad. The median is “6: agree,” and for two indicators it is “7: strongly agree.” For all variables, the distribution of the data are negatively skewed – that is, the curves tend to the right – and participants strongly agreed that street vending was highly pervasive in Baghdad during September–November 2018. The final column of Table 1 shows the outer loadings or correlations with the construct of street vending. If the value is greater than the cut-off value of 0.7, an item should be considered acceptable and reliable. If the outer loading is less than 0.4, such as that for SV1, it should be omitted and considered as invalid. If it is between 0.4 and 0.7 and the validity and reliability of the construct are guaranteed, it should be retained. Four variables (SV2–SV5) are reliable and can be used to reflect street vending.

### Poverty

Studies consider poverty a primary reason for the existence of street vending. Poverty is associated with political instability, civil war, recession, and economic crises, and increases when there is low economic growth, weak economic development, and income inequality. Less-developed countries are characterized by high population growth and large family sizes. In addition, Agricultural problems drive migration from rural areas to cities because agriculture suffers from limited infrastructure and low productivity. In short, these migrants do not have the financial resources to run formal businesses.

Generally speaking, adolescents and young children work in public streets in harsh conditions. And the public considers their SVs deviant and low status. These young people face disgrace, abasement, and stigma. Their dignity is frequently damaged. Half of the female SVs in Johannesburg, South Africa, for example, lift heavy items and struggle with injuries such as cuts, burns, and musculoskeletal problems. These females work without appropriate stalls in dirty, noisy, and crowded markets. Additionally, they work in unsafe environments, subject to verbal abuse and even violence. Finally, peddlers are exposed to air pollutants, while they increase the problem of waste (Pick et al., 2002).

Since poverty results in the existence of street vending, it is expected that the latter will disappear as the economy develops. In recent years, the financial resources of the Iraqi government have decreased because of the sharp decline in oil prices. Moreover, the limited resources have mostly gone to the military to fight terrorism. As a result, the state has stopped recruiting youth and graduates. Consequently, street vending has increased due to rising unemployment and poverty. In general, low-income people lack adequate savings and do not have access to formal credit, so they cannot expand their businesses or transition into the formal sector.

The above discussion provides tools to build a measurement model for poverty. It can be measured by the large size of low-income families; the drop in agricultural production; the inability of peddlers to expand their businesses; young people earning money in the informal sector; and the experience of being subject to dirt, noise, and abuse. The dataset for poverty has a similar pattern to that for street vending. If we rely on the median as a central tendency, as explained above, participants agree on the high level of poverty among SVs or within society, as shown in Table 2. For some indicators, Iraqis strongly agree on the drop in agricultural production and the harsh environment in which peddlers operate. The data do not have a normal distribution: it deviates to the right, or the best normal curve is negatively skewed because the mode is mostly at “7: strongly agree.” All indicators have outer loadings above the cut-off point of 0.7 – that is, the indicators have acceptable correlations with the underlying construct. Only PO3 (SVs cannot expand their business) performs poorly. However, the reliability and validity of the construct have been achieved. Thus, the indicator of PO3 is also reliable, with the result that the measurement model of poverty is guaranteed.

### A lack of education

A lack of education is another construct explaining the existence of street vending. Generally, it is hard for semi-educated and illiterate people to get jobs in the formal sector, so they have to work in the informal sector in order to survive. Lack of qualifications is a significant obstacle to employment, as is a lack of experience even for educated people. Street vending is an easy solution for such people to earn money. Many SVs have primary or secondary certificates, but usually, they do not have a university degree. As people become more educated, they may perform better even when working in the informal sector.

Even Iraq has been involved in successive wars since 1980, the most disastrous was in 2003 when the United States-led alliance occupied the country which results that the state and its infrastructure collapsed. Thereafter, Iraq struggled from civil war, terrorism, and sectarian tensions. Accordingly, the educational system suffered excessively crowded classrooms and three shifts per day for each school. And the government barely paid staff’s salaries and it did not allocate little funds for capital expenses. That leads to (1) school dropouts increased and the effectiveness of education declined, (2) schools and universities produce thousands of graduates with poor practical and technical skills and few entrepreneurial skills. This creates difficulties for graduates to obtain employment or start small businesses. Students who leave school early work on the streets with some of the fresh graduates. Additionally, poverty increases the number of dropouts from schools, with families sending their children to work in the informal sector (Dyer, 2007).

According to researchers, lack of education can be reflected by observed variables; for example, SVs are illiterate or semi-educated; they do not have appropriate qualifications or university degrees to get jobs in the formal sector; students increasingly drop out from schools, and the educational system does not improve the employability of graduates. Table 3 shows that the median is close to “5: somewhat agree,” although participants agreed or strongly agreed about school dropouts and the low effectiveness of the educational system. The data are negatively skewed or the curve deviates to the right with a long (flatter) tail to the left. This indicates that participants agreed on a weak educational system in which graduates cannot get jobs easily in the formal sector or start small businesses. All indicators are reliable since the outer loadings exceed the threshold of 0.7, and/or the construct guarantees reliability and validity. The measurement model is acceptable since no indicator scores less than 0.4.

### Immigration

Street vending can be explained by immigration. In general, civil wars and conflicts drive people to flee from their cities to relatively safe ones. The easiest way for them to survive is to run a business on the streets with a tiny amount of financing. Terrorism has recently affected the northern cities of Iraq, with the result that some migrants have escaped to Baghdad and occupied the sidewalks to sell fruit and vegetables. Historically, and especially when there was a boom in oil prices, low-income people have migrated from southern Iraq, particularly from rural areas, to Baghdad where they were able to enjoy a higher level of development, better infrastructure, and, therefore, more opportunities. The stabilization of the economic and political system in South Africa, for example, attracted migrants from surrounding countries. Thus, the number of SVs in South Africa doubled from 1993 to 2002 and then tripled between 2002 and 2007 (Tengeh and Lapah, 2013).

Some migrant SVs successfully operate their businesses and introduce new recipes for traditional meals. However, they do not have proper documentation to stay in new places, and their children accompany them. They face harsh eviction by the municipal authorities and the police. Moreover, they do not have sufficient financial resources to start formal businesses and obtain licenses that require a long bureaucratic process. In short, conflicts lead to immigration, and the latter increases the spread of street vending.

Poverty negatively impacts the effectiveness of educational attainment and low-income families do not have the resources to register their children in private schools or to afford the required books and stationery. Thus, school dropouts are much higher among low-income people who prefer their children to work and earn money. Since semi-educated people do not have the qualifications to get jobs in the less-developed cities, this gives them an incentive to move to better places in search of work and a better standard of living. Those migrants from poor and rural areas face difficulties to upgrade their skills in schools and universities. This is another reason, therefore, why they leave education and work on the streets at younger ages.

To build a measurement model for immigration, it is necessary to find measurable variables that reflect the attitudes of the general public. Attitudes can be tracked when the public sees some migrant SVs working in their cities and when those peddlers come from poor, rural areas or cities associated with conflicts and civil wars. Some migrants are women and children who work informally. Additionally, some migrants manage to start successful businesses even on the streets and with tiny resources. Table 4 shows that participants chose “5: somewhat agree” or “6: agree” in response to the statement about the existence of migrant SVs, since most vendors were from the same city as the participants. For this reason, participants did not strongly agree that the SVs are migrants. The distribution of data are negatively skewed to the right, which indicates participants’ acceptance of the existence of migrant SVs. Most indicators have outer loadings above the cut-off point of 0.7. The indicator of IM5 is also acceptable because the construct achieved reliability and validity.

### Unemployment

Street vending also stems from unemployment. It is expected that the unemployment rate is higher in developing countries than in developed countries because the ratio of investment to gross domestic product is much lower in the former due to low income per capita. The duration of unemployment is also longer in developing countries because of their rigid economies and limited technological advances. Unemployed people work as SVs for survival even though their productivity and earnings are limited. The rural sector does not offer vacancies. Therefore, people migrate to cities where they work in the informal sector because they cannot get jobs in the formal sector due to their limited qualifications and experience (Truong, 2018).

It is worth noting three examples: (1) Unemployment triggered extensive protests in July 2018 in Baghdad along with cities in the south of the country. Protesters criticized high unemployment, corruption, and failing electricity and water supply infrastructure. (2) When the global financial crisis affected developing countries, some laid-off employees start their own informal businesses using their experience and entrepreneurial skills. And (3) the high unemployment due to the recent coronavirus pandemic will likely result in an increase in the number of SVs. Shortly, poverty and recession lead to high unemployment, and the latter increases the prevalence of street vending.

Semi-educated unemployed people cannot easily get jobs in the formal sector, which reduces the incentives for low-income people to get higher certificates. If the public officials evict SVs, demand for jobs will increase and the problem of unemployment will be much higher than it is. A weak educational system does not equip students with up-to-date skills. Therefore, graduates’ employability is limited, and employers cannot benefit from graduates able to significantly increase output and competitiveness. When poverty was associated with conflicts in northern Iraq, semi-educated people, especially from rural areas, emigrated to Kurdistan, Iraq, or illegally to Turkey and Europe. Such migrants increase the unemployment rate in host cities or countries. The migrants do not have adequate financial resources to start formal businesses and to obtain the documents and licenses required to work formally. Therefore, they have to survive by working in the informal sector, primarily as SVs. To conclude, economic immigration is when people suffer high unemployment in their local areas and emigrate to improve their lives and find jobs in other places.

In the context of this paper, unemployment can be measured by getting public responses to some statements, such as unemployment is high among youth and fresh graduates; the government does not provide unemployment compensation; the duration of being unemployed is long; the rural sector does not offer vacancies, and street vending is a solution for survival. Table 5 shows that, with respect to the median, most participants chose “7: strongly agree” when responding to statement about the existence of high unemployment. The mean, which is affected by low scores, indicates that respondents agreed that unemployment is clearly high among youth and fresh graduates. The distribution of the data are negatively skewed and not normally distributed. This suggests that participants most likely strongly agreed on the existence of extensive unemployment. All observed variables are reliable since outer loadings exceed the cut-off point of 0.7, or the reliability and validity of the construct of unemployment are guaranteed. Thus, the measurement model for unemployment is acceptable and can be used in further studies.

### Urban culture

Urban culture leads to the pervasiveness of street vending and comprises the values and practices common among citizens. Street vending is a crucial part of street culture and peddlers or hawkers give vibrancy to cities. Baghdad is different from Cairo, Egypt. In general, people love their cities because each city offers its own goods, services, and activities. The urban culture of a particular city cannot be found elsewhere. Some SVs spend most of their lives working on the streets and do not have a desire to shift to the regulations and policies of the formal sector. Moreover, they prefer freedom and flexibility to the bureaucratic system. Additionally, they consider their jobs as professions and have spiritual incentives to stay in public spaces, which lead them to be satisfied and accordingly to try to satisfy their customers (Wibisono and Catrayasa, 2018). On the other hand, customers spend a couple of hours per day visiting the closed traditional markets where SVs occupy the sidewalks. They visit public spaces not only to make consumer purchases but also to spend their leisure time and to communicate with their friends. Therefore, they love walking there in the same way as people enjoy visiting a park.

Peddlers prefer their professions to putting themselves in a concrete office akin to a prison. Consequently, it is understandable why they put up with the hazardous environment rather than the safety provided by formal businesses. They love flexible and open spaces. Furthermore, they carry their hopes while exploring new spaces. In addition, they try to change the location of their stalls to be closer to their customers. Some vendors inherit this profession from previous generations, and in general, it reflects their social values and lifestyle. Moreover, the urban culture determines the cuisine and eating habits of a particular city’s inhabitants. Customers, on the other side, share similar values to those of the hawkers. It is easier to buy a newspaper from kiosks than to go to bookshops. Al-Mutanabbi street is a historical place in Baghdad where peddlers occupy sidewalks in front of publishers and shops offering new and old books. Readers always visit this beautiful place where intellectuals give poetry recitals and cultural presentations to visitors. All activities operate in open places along the street.

To build a measurement model for urban culture as viewed by the public, we can derive some measurable variables based on the literature: the public recognizes the city as vibrant, with the pervasiveness of SVs offering traditional meals as a contributing factor to this vibrancy; peddlers love their professions; customers spend leisure time in crowded traditional markets; and it is possible to find unique books in a special street such as Al-Mutanabbi in Baghdad. Table 6 demonstrates that, based on the median parameters, most respondents chose “5: somewhat agree” on the existence of urban culture associated with the existence of SVs. The data are negatively skewed to the right with a long tail to the left as all skewness parameters are negative. This represents participants’ attitudes toward the presence of urban culture. The observed variable UC3 should be omitted since the outer loading did not meet the threshold value of 0.7. Other variables are reliable and can be used to measure and reflect the urban culture.

### Low-income consumption

Low-income consumption is another factor affecting the existence of street vending in which SVs offer cheaper products than retail shops do because they do not incur some operational costs, such as license fees, electricity supply, and rents. Customers who enjoy walking on the sidewalks are attracted by peddlers even though the SVs cannot compete with retail shops on brands, quality, and variety of products where crowded traditional markets combine both SVs and retail shops. Although the food sector is controlled by modern restaurants, hawkers provide traditional and delicious alternatives. Customers unable to afford expensive food prefer purchasing from the streets, and peddlers try to allocate their business to nearby customers and respond to a changing environment. As an evident, tourists normally like to buy souvenirs with attractive designs and at cheap prices from SVs.

Street vendors are sometimes blamed for trading pirated goods. However, this criticism might be extended to formal retailers. Peddlers do not stick to formal working hours. Thus, they can respond to customers’ needs at appropriate times. This has resulted in some peddlers being innovative or creative, leading them to earn good money despite their limited education. In recent years, informal vendors and home-based small businesses have interacted with customers through social media such as Facebook and WhatsApp, especially those businesses that offer maintenance and construction services.

Consumers realize that purchasing from public streets is part of their urban culture where they have loyalty to their cities. For instance, Iraqis love eating meat-based meals at any time, even for breakfast. SVs are well known in Baghdad for offering delicious *kabak*, *tekka*, and *bachah*, all of which are meat dishes. In general, food consumption habits are affected by geographical locality, the range of fauna and flora, and climate. Moreover, the consumption pattern is inherited from previous generations, and Meat consumption is sensitive to religion, history, and local culture (Nam et al., 2010). The urban culture teaches vendors to produce delicious food and drink. For example, Iraqis love drinking hot black tea all day. Thus, all traditional markets in Baghdad include plenty of tea sellers. In short, the street culture establishes exchange channels between trusted sellers and low-income consumers, and the pattern of low-income consumption creates a demand for goods offered by SVs.

We can record the attitudes of the public toward low-income consumption patterns by some measurement items: SVs are near to customers; fruit and vegetables are cheaper at street sellers than at retail shops; peddlers offer delicious and cheap cooked food; souvenirs and accessories are available at low prices in the streets; and hawkers offer similar products to retail shops. Table 7 presents the dataset for the low-income consumption construct. According to the parameters of the median, respondents chose “5: somewhat agree” on the presence of a low-income consumption pattern. The data are negatively skewed to the right with a long tail to the left – that is, the mode is greater than the median, which is clear evidence of agreement about the existence of such a pattern. The first indicator LC1 should be omitted because it does not meet the statistical requirements. All other measurable variables can be considered reliable because the outer loadings exceed the threshold value of 0.7 or the reliability and validity of the construct are guaranteed.

### Resistance

Street vending also stems from resistance. If the municipalities and traffic police reclaim pedestrians’ pavements by demolishing SVs’ stalls, they may succeed in removing SVs and their stalls and products. As a result, the vendors become unemployed and therefore try to develop strategies to deal with the eviction that harms their livelihood. Moreover, peddlers can take drastic action and become resistant when the police implement restrictive regulations to control the sidewalks. And, citizens and shoppers often support poor hawkers who come back to their sites after eviction with more determination. In general, SVs rely on their social networks when they occupy certain public places or they have close relationships with relatives and friends in a specific community. This provides them with a sort of power against retailers, pedestrians, and public authorities. Peddlers always resist being removed from staying on the pavements in the traditional markets. They sometimes develop individual and collective responses such as planned and deliberate protests. Moreover, they probably pay bribes to officials so that they can be allowed to stay on the sidewalks (Lata et al., 2019).

Generally, SVs do not have a representative organization such as a union. Therefore, they usually lack collective power when a situation requires negotiation with public entities, such as municipal authorities, police, and traffic police. However, they adopt strategies to enable them to have a kind of formal or informal power according to the circumstances in a specific city. And, they try to produce their own leaders or to build ties with key tribal figures or even with some officials who are collaborative with and sympathetic toward them. Since eviction threatens their livelihood, the SVs strongly resist being removed from sidewalks and traditional markets regardless of the concerns of public authorities, formal shops, and residents. They argue that their informal businesses are the only means of survival; otherwise, the government should guarantee them jobs irrespective of their qualifications. Moreover, poor unemployed people develop a subculture to cope with oppression. In other words, the poor community develops its own oppositional cultures that help it to reject the public trends in which peddlers believe that they have the right to survive in their areas, and hence that public officials do not have the right to evict them and leave them without a minimum level of income.

Unlicensed SVs strive to work and remain in the crowded traditional markets. Accordingly, Resistance can be measured by several variables: peddlers resist eviction from sidewalks and traditional markets; they develop strategies to enhance their ability to stay in the streets; they return to their sites after public officials have demolished their stalls; they sometimes protest against eviction campaigns; and they depend on their social networks to occupy certain open places. Table 8 shows that, according to the parameters of the median, respondents clearly chose “6: agree” in relation to the presence of resistance among SVs. The mean is also close to “6: agree” with respect to the reality of resistance. The distribution of data are negatively skewed to the right with a flatter tail to the left – that is, the data deviate into “7: strongly agree” on the existence of resistance. The mode is at “7: strongly agree,” which reflects the most frequent answers to the measurable variables. All indicators are clearly reliable because the outer loadings are higher than the threshold value of 0.7. We can safely depend on these indicators as a way of reflecting resistance.

### A lack of microfinance

A lack of microfinance is another factor explaining the existence of street vending. Generally, SVs rely on their own personal savings to finance their micro-businesses, which require a little money. They regularly tend to sell their valuables, such as furniture and jewelry. If they have nothing, which is the case for many poor and unemployed people, they borrow funds from informal channels at abnormal interest rates because they are unable to obtain credit from financial funds or commercial banks (Husain et al., 2015). The government should create special organizations to finance such micro-businesses. Furthermore, commercial banks should lend to peddlers based on the peddlers’ knowledge of the markets and as a socially responsible practice, rather than making a decision based on a technical evaluation of the financial risk.

Hawkers cannot afford to start formal small businesses, or to pay rents and the expenses of getting licenses because they are the poorest category in society. Therefore, they stick to these difficult jobs because they are unable to expand their business or to shift to a formal business. They do not have the financial documentation necessary to apply for loans, while commercial banks themselves do not enter into collaboration with poor people. A distinct lack of microfinance pushes poor unemployed people to survive by working on the sidewalks of the traditional markets. However, a developed financial system could offer suitable loans to youth and unemployed people to start formal small businesses. This practice should be supported and supervised by governmental entities. To conclude, SVs have to continue working in the public spaces and bearing the difficulties of such jobs because they cannot access the financial system.

Based on the extant literature, the measurement model of lack of microfinance can be reflected through some observed variables: SVs cannot access formal credit facilities; they rely on their limited savings and by selling their valuables; they pay abnormal interest rates to borrow from informal channels; they cannot rent shops or expand their businesses; and the government does not financially support micro-businesses. Table 9 displays public attitudes toward the lack of microfinance. The parameters of the median show that respondents mostly chose “6: agree” on the existence of this problem. Although the mean is affected by low scores, it is located within the area of agreement. The data are negatively skewed to the right with a long tail to the left, because the skewness parameters are all negative. This is evidence that the spread of data deviates to a strong agreement on the lack of microfinance in Baghdad. The indicators are reliable since the outer loadings are above the threshold value of 0.7 or the reliability and validity of the construct are guaranteed as revealed by the statistical results of PLS-SEM (Al-Jundi et al., 2020).

The mean of all constructs’ indicators is close to 5.5 (between “5: somewhat agree” and “6: agree”). Since the sample standard deviation is close to 1.5, we can state that the spread of most points is in the range from “4: neither agree nor disagree” to “7: strongly agree.” The participants most likely agree on the existence of street vending and the main factors driving it. The mode is close to strong agreement on the presence of the problem.

Using the current dataset, the researchers found that poverty significantly impacts street vending. We also investigated the interplay among variables and concluded the following: weak educational system and high unemployment mediate the impact of poverty on street vending; internal migration and high unemployment mediate the impact of poverty on street vending; and, in the most important path, the weak educational system, internal migration, and high unemployment sequentially mediate the impact of poverty on street vending (Al-Jundi et al., 2020). We additionally found, on another project, that resistance mediates the impact of urban culture on street vending; a low-income consumption pattern and resistance sequentially mediate the impact of urban culture on street vending; and resistance again mediates the impact of the lack of microfinancing on street vending. However, the direct effect of urban culture on street vending is not significant, while the lack of microfinancing positively influences the spread of operating businesses on the sidewalks of traditional markets (Al-Jundi et al., 2022). The dataset can be used to examine the relationships between constructs as suggested by the theoretical justification. Additionally, the measurement models can be implemented to study the interplay among these constructs in other cities.

## Value of the data

1. The survey dataset, which shows the perception of the general public in Baghdad, Iraq, is complete and usable by researchers around the world, especially those in developing countries where street vending is highly pervasive.
2. The dataset comprehensively measures the attitudes of ordinary people toward the issue of street vending and the main factors driving it. Thus, it can serve as a basis for revisiting public policies to fairly deal with street vendors.
3. The dataset and its code are genuine and present a reasonably large sample size. It is ready to be used by researchers applying a partial least squares/structural equation modeling approach.
4. The dataset introduces novel and rich measurement models of street vending and the main factors driving it (poverty, lack of education, immigration, unemployment, urban culture, low-income consumption, resistance, and lack of microfinance).
5. The dataset can be easily reviewed and downloaded. And researchers can use the same measurement items (the questionnaire) to gather data for other countries and make comparisons with the current experience of street vending in Baghdad, Iraq.

## Data availability statement

The original contributions presented in the study are included in the article/supplementary material. Supplementary data to this article can be found online at https://doi.org/10.17632/dh3cv5p7rv.1. The Arabic version of the questionnaire can be found online at https://forms.gle/YrdHM44E1ayfCTB6A.

## Ethics statement

Ethical review and approval was not required for the study on human participants in accordance with the local legislation and institutional requirements. Written informed consent for participation was not required for this study in accordance with the national legislation and the institutional requirements.

## Author contributions

All authors listed have made a substantial, direct, and intellectual contribution to the work, and approved it for publication.
